# Extra-Uterine Pregnancy

**Published:** 1893-01

**Authors:** Edwin Ricketts

**Affiliations:** Cincinnati, Ohio


### ABSTRACT—EXTRA-UTERINE PREGNANCY.

BY DR. EDWIN RICKETTS, CINCINNATI, OHIO.

Simplest classification is following, by Heywood Smith:

1st. Pre-ruptured stage.

2nd. Ruptured stage.

3rd. Post-ruptured stage.

Primary rupture is, in majority of cases, tubal.

Positive diagnosis of extra-uterine pregnancy in pre-ruptured stage is rarely possible.

Rupture after third month more disastrous than previous to that time.

Dr. R., operated successfully during the eighth week—the earliest operation he had ever seen performed.

Majority of cases rupture into peritoneal cavity; these are more serious than those that rupture into the broad ligament.

Dr. R. also reports a case when fœtus died at sixth month and bony skeleton was delivered per rectum; patient was confined to bed for two years but recovered. An early abdominal section in this case could not have been more serious.

If operators and expert diagnosticians cannot diagnose in pre-ruptured stage, how can so-called electricians pronounce cures in pre-ruptured stage by electrolysis?

Cannot hope to do good in ruptured stage with electrolysis.

Great majority, some claim that all cases of intra-peritoneal rupture, die without operation.

Treatment—operation, with clean knife, clean hands, clean water and clean ligations, and that, too, as early as possible.

---

THE DANGERS OF TATTOOING.—Dr. Liebreich reports the case of a soldier who, after being tattooed upon both arms, developed tuberculosis of the lungs. He was of a healthy family and had always been well. The writer describes the process of tattooing, and mentions the possible dangers of infection from the saliva of the tattooer. Severe hæmorrhages, lymphangitis, phlegmonous processes, and especially syphilis have been known to follow tattooing. First sailors, then masons, carpenters, and soldiers are most frequently found tattooed.—*Cleveland Med. Gaz.*

